# Modulation of Inflammasome Activity by miR-197-3p in Familial Mediterranean Fever Mouse Macrophages

**DOI:** 10.5152/ArchRheumatol.2026.25179

**Published:** 2026-04-03

**Authors:** Yeliz Z. Akkaya-Ulum, Basak Sen, Tayfun Hilmi Akbaba, Banu Balci-Peynircioglu

**Affiliations:** Department of Medical Biology, Hacettepe University Faculty of Medicine, Ankara, Türkiye

**Keywords:** Autoinflammation, Familial Mediterranean fever, macrophages, miR-197-3p, pyrin inflammasome

## Abstract

**Background/Aims:**

Familial Mediterranean fever (FMF) is an autosomal recessive inflammasomopathy caused by mutations in the *MEditerranean FeVer* (*MEFV*) gene and is characterized by recurrent inflammatory attacks largely associated with pyrin inflammasome activity. Although FMF is a monogenic disease, clinical heterogeneity suggests the contribution of additional regulatory mechanisms, including epigenetic factors such as microRNAs (miRNAs). Previous studies identified miR-197-3p as a regulator of inflammatory signaling by targeting *IL1R1*. The objective of this study was to investigate the functional role of miR-197-3p in pyrin inflammasome activation and priming mechanisms in macrophages derived from an FMF knock-in mouse model.

**Materials and Methods:**

Bone marrow–derived macrophages (BMDMs) from *Mefv*^V726A/V726A^ mice were transfected with pre–miR-197-3p or a mimic control. Following transfection, inflammasome priming and activation were induced using specific inflammasome activators and Toll-like receptor (TLR) agonists targeting pyrin, AIM2, and NOD-like receptor protein 3 pathways. Interleukin-1β (IL-1β) secretion and pro–IL-1β expression were subsequently assessed.

**Results:**

Overexpression of miR-197-3p significantly reduced IL-1β secretion following just pyrin inflammasome activation, while responses mediated by other inflammasome pathways were largely preserved. In addition, miR-197-3p overexpression was associated with decreased pro–IL-1β expression in response to TLR2 stimulation.

**Conclusion:**

These findings suggest that miR-197-3p may preferentially influence pyrin inflammasome–related inflammatory responses in FMF macrophages. While the present data do not establish a direct mechanistic interaction, they support a potential regulatory role for miR-197-3p in FMF-associated inflammation and provide a basis for future mechanistic and in vivo studies.

**Main Points**

- miR-197-3p overexpression was associated with reduced interleukin-1β (IL-1β) release, most prominently following pyrin inflammasome activation.
- Pro–IL-1β expression was altered in the presence of miR-197-3p during Pam3 and HKLM stimulation.
- Stimulation with specific Toll-like receptor (TLR) agonists, particularly TLR2 activators, was associated with reduced pro–IL-1β expression in miR-197-3p–overexpressing cells.
- The findings suggest a preferential association between miR-197-3p expression and pyrin inflammasome–related inflammatory responses in familial Mediterranean fever knock-in mouse macrophages.

## Introduction

Familial Mediterranean fever (FMF) is an autosomal recessive inflammasomopathy caused by hypermorphic mutations of *MEditerranean FeVer (MEFV)* gene.^1^ Recent studies revealed that pyrin-associated autoinflammatory diseases comprise a group of rare genetic disorders resulting from mutations in the *MEFV *gene, which encodes the pyrin protein. Pyrin plays a critical role in innate immune cells, particularly neutrophils and macrophages, by regulating inflammasome activation. Dysregulated pyrin inflammasome activity leads to excessive interleukin-1β (IL-1β) production, which contributes to the recurrent inflammatory attacks characteristic of FMF.^2^^,^^3^

Although FMF is a monogenic autoinflammatory disease, variations in clinical presentation among patients with the same genotype and the presence of active disease in *MEFV *mutation carriers make it difficult to establish a clear phenotype-genotype correlation.^4^^-^^6^ This observation suggests that additional regulatory mechanisms, including epigenetic factors, contribute to disease pathogenesis. Among these, microRNAs (miRNAs) have emerged as important post-transcriptional regulators of inflammatory signaling pathways and inflammasome activity.^7^

Recent studies have reported altered miRNA expression profiles in FMF patients.^8^^,^^9^ In previous work, significantly reduced expression of miR-197-3p was identified in homozygous FMF patients, and it was demonstrated that miR-197-3p directly targets the interleukin-1 receptor type I (IL1R1), thereby modulating IL-1β–dependent inflammatory signaling.^10^ However, whether miR-197-3p directly influences inflammasome activation—particularly the pyrin inflammasome central to FMF pathogenesis—remains unclear.

Innate immune priming through Toll-like receptors (TLRs), especially TLR2, plays a key role in inflammasome activation by inducing pro–IL-1β expression via NF-κB signaling. Increased TLR2 expression has been reported in immune cells from FMF patients, suggesting a functional link between TLR-mediated priming and FMF-associated inflammation^11^ and monocytes.^12^ Although emerging studies suggest a potential association between TLR2 signaling and FMF-related inflammatory pathways, the interaction between miR-197-3p, TLR signaling, and pyrin inflammasome activation has not yet been systematically investigated. Therefore, the precise contribution of these pathways to FMF pathogenesis remains to be fully characterized.

Chae et al^13^ showed moderate similarity in the amino acid sequence between the human and mouse *MEFV* genes (with a similarity of 65.5%). Further study showed that in FMF “knockin” mice carrying mutant human B30.2 domains, gain-of-function pyrin mutations (*Mefv*^V726A/V726A^) lead to an autoinflammatory disease through an ASC-dependent inflammasome mechanism that is independent of NOD-like receptor protein 3 (NLRP3).^14^ The FMF KI mouse model exhibits several key phenotypes that closely mimic the disease in humans. These include growth retardation, wasting, dermatitis, and arthritis, alongside more typical FMF characteristics.

Although the previous studies demonstrated that miR-197-3p regulates inflammatory signaling in human FMF peripheral blood mononuclear cells by directly targeting *IL1R1*, these findings were limited to receptor-level modulation and did not address inflammasome-specific mechanisms. In particular, the potential involvement of miR-197-3p in pyrin inflammasome activation—the central inflammasome implicated in FMF pathogenesis—has not been systematically investigated. To date, miR-197-3p has not been examined in pyrin inflammasome–driven experimental models, nor has its possible role in inflammasome priming and activation been evaluated in a disease-relevant FMF knock-in system. Therefore, whether miR-197-3p is associated with alterations in IL-1β production through modulation of pyrin inflammasome–related pathways, rather than through general inflammatory signaling, remains unclear.

To address this gap, the present study aimed to explore the effects of miR-197-3p overexpression on inflammasome priming and activation in macrophages derived from an FMF knock-in mouse model (Mefv^V726A/V726A^). Specifically, the association between miR-197-3p expression and pyrin inflammasome–related IL-1β secretion was investigated, and it was evaluated whether these effects differed from those observed in other inflammasome activation pathways.

## Materials and Methods

### Reagents

Pam3Cys-Ser-(Lys)4, TLR1/TLR2 agonist (tlrl-pms); Class A CpGODN 2395, TLR9 agonist (tlrl-2395); FSL-1, TLR2/TLR6 agonist (tlrl-fsl), heat-illed Listeria monocytogenes (HKLM), TLR2 agonist (tlrl-hklm), and GQ single-stranded RNA (ssRNA) PolyU LyoVec, and TLR7 agonist (tlrl-lpu) were from InvivoGen. Flagellin (tlrl-pstfla) and lipopolysaccharide (LPS) (tlrl-pelps) were obtained from InvivoGen. C3 toxin (CT03) was from Cytoskeleton. Adenosine 5’-triphosphate disodium salt (ATP) (tlrl-atpl) was obtained from InvivoGen.

### Cell Preparation and Culture

Bone marrow cells from 6- to 12-week-old male and female FMF-KI mice (Mus musculus) on a C57BL/6J background, originally generated by Dr. Jae Jin Chae and Dr. Daniel L. Kastner (NHGRI, NIH), were provided for this study. All animal procedures were approved by the Hacettepe University Local Ethics Committee for Animal Experiments under the registration number 2023/07 and decision number 2023/02-02 (March 14, 2023) by the institutional animal care committee. Informed consent was not applicable because this study involved animal experiments. All animal procedures were approved by the Institutional Animal Care and Use Committee (IACUC) / Local Ethics Committee and were conducted in accordance with institutional guidelines. Male and female mice were included in all experiments in equal numbers, and data were pooled, as no sex-specific differences were observed under the experimental conditions. Importantly, littermate controls were used throughout the study, ensuring that mutant and control animals shared the same genetic background and prenatal environment. Chae et al^13^ previously described FMF-KI mice harboring an FMF-associated mutant human B30.2 domain (*Mefv*^V726A/V726A^).

Bone marrow progenitors from the tibia and femur of 6-12-week-old male or female mice were used to generate bone marrow–derived macrophages (BMDMs). After isolation, BMDMs were frozen in freezing medium (heat-inactivated fetal bovine serum (FBS, Invitrogen) with 10% dimethyl sulfoxide (Merck)). Frozen cell stocks were gently thawed and maintained in Iscove’s Modified Dulbecco’s Medium supplemented with 20 ng/ml M-CSF (PeproTech), 10% heat-inactivated FBS (Invitrogen), 1 mM sodium pyruvate, and antibiotics (100 U/mL penicillin and 100 μg/mL streptomycin; Invitrogen) for 7 days. Subsequently, BMDMs were seeded into 12-well plates 24 hours prior to stimulation.

The BMDMs were transfected with 20 nM hsa-miR-197-3p mimic (mirVana, Ambion) or a negative control mimic (mirVana miRNA Mimic Negative Control #1, Ambion) using 3 μL Lipofectamine 2000 (Invitrogen) following the manufacturer’s protocol.

### Inflammasome Activation and Priming

BMDMs (1.5 × 10^6^ cells per well) were plated in 12-well plates in RPMI 1640 (Invitrogen) containing 10% FBS and antibiotics. On the day of pre-miR-197-3p and mimic control transfection, the medium was changed to Opti-MEM (Invitrogen) and transfection was performed. After 24 hours, the transfection medium was changed to complete RPMI 1640. Cells were incubated for a total of 48 hours. The BMDMs were stimulated with 1 μg/mL LPS in Opti-MEM (Invitrogen) for 3 hours. Then, the cells were collected for miRNA isolation and immunoblot. Supernatants were used for enzyme-linked immunosorbent assay (ELISA).

Following 3 hours of LPS priming, inflammasome activation was induced using specific stimuli. Pyrin inflammasome activation was triggered by treating the cells with C3 toxin (0.5 μg/mL) for 6 hours. To activate the AIM2 or NLRC4 inflammasomes, cells were incubated in Opti-MEM containing either 1.25 μg/mL dsDNA complexed with Lipofectamine 2000 (3 μl/mL; Invitrogen) for 40 minutes or 0.5 μg/mL flagellin mixed with 30 μl/ml DOTAP (Roche) for 1 hour, respectively. For NLRP3 activation, the culture medium was replaced with RPMI 1640 supplemented with 5 mM ATP and incubated for 30 minutes. After stimulation, culture supernatants were harvested for ELISA analysis. There aren’t any conditions that prevent the activation of different inflammasomes. The BMDM cells respond well to different activators of inflammasomes after LPS priming.

Bone marrow–derived macrophages were seeded at a density of 1.5 × 10^6^ cells per well and primed with the indicated TLR ligands: LPS (1 μg/mL), Pam3CSK4 (Pam3, 1 μg/mL), CpG oligodeoxynucleotide (CpG ODN, 100 μg/ml), FSL-1 (0.5 μg/μL), heat-killed *Listeria monocytogenes* (HKLM, 1 × 10^10^ particles/mL), or GQ single-stranded RNA (GQ ssRNA, 1 μg/μL). Priming was performed for 3 hours in RPMI 1640 medium (Invitrogen) supplemented with 10% FBS and antibiotics. Following priming, culture supernatants were discarded, and cell pellets were harvested for immunoblot analysis. Cell viability was monitored under all experimental conditions, and none of the reagents used in the priming assays exhibited detectable cytotoxic effects.

### RNA Isolation and Quantitative Reverse Transcription Polymerase Chain Reaction

RNA isolation was carried out using the miRNeasy Mini Kit (Qiagen) from pre-miR-197-3p and mimic control transfected cells. The expression analysis of miR-197-3p miRNAs was done by RT-qPCR. The TaqMan® MicroRNA Reverse Transcription Kit (Applied Biosystems) was used to do the reverse transcription. All reactions were carried out using MicroRNA TaqMan® hsa-miR-197-3p, 000497 and U6 snRNA, 001973 assays (Applied Biosystems). The reactions were conducted on the BioRad IQ5 and Applied Biosystems ViiA 7.

### Immunoblot and Enzyme-Linked Immunosorbent Assay

The immunoblots were created using Invitrogen’s Novex® Tris-Glycine Gel Systems and probed with the following antibodies overnight at 4°F: anti-mouse IL-1 antibody (AF-401-NA); anti-ASC antibody (sc-22514-R); anti-NLRP3 antibody (AG-20B-0014-C100); anti-pyrin antibody (Ab93, produced by XXX); and anti-actin (sc-1615, Santa Cruz Biotechnology).

Levels of secreted IL-1β in the culture supernatants were quantified using a commercial ELISA kit (Quantikine Mouse IL-1β/IL-1F2 ELISA Kit, MLB00C; R&D Systems) according to the manufacturer’s protocol.

### Statistical Analysis

Differential expression of the miR-197-3p was normalized according to RNA, *U6 Small Nuclear 1* (*RNU6*). Relative gene expression levels were determined using the 2^−ΔCt^ method. For western blot analysis, *β-actin* was used as a normalizer. In this study design, the group to be compared with pre-miR-197-3p is the mimic control group. Non-transfected cells and Lipofectamine 2000 are used solely as experimental controls. Pairwise comparisons were initially considered with respect to the mimic control group. Therefore, Student’s *t*-test was used for the pairwise comparisons since the data follow a normal distribution. In addition, ordinary one-way ANOVA analysis was also performed. The analyses were conducted accordingly, and the results were consistent, with the same significant findings emerging. Student’s *t*-test and ordinary one-way ANOVA were statistically analyzed for all experimental set-ups using GraphPad Prism 8.0, version 8.3.0 (538) software (https://www.graphpad.com/) ()(GraphPad Software LLC; San Diego, CA, USA). This test was applied to all relevant data where comparison between 2 groups was necessary. *P*-values less than .05 were regarded as significant. All the experiments were performed with 3 biological replicates.

## Results

**Figure 1.**
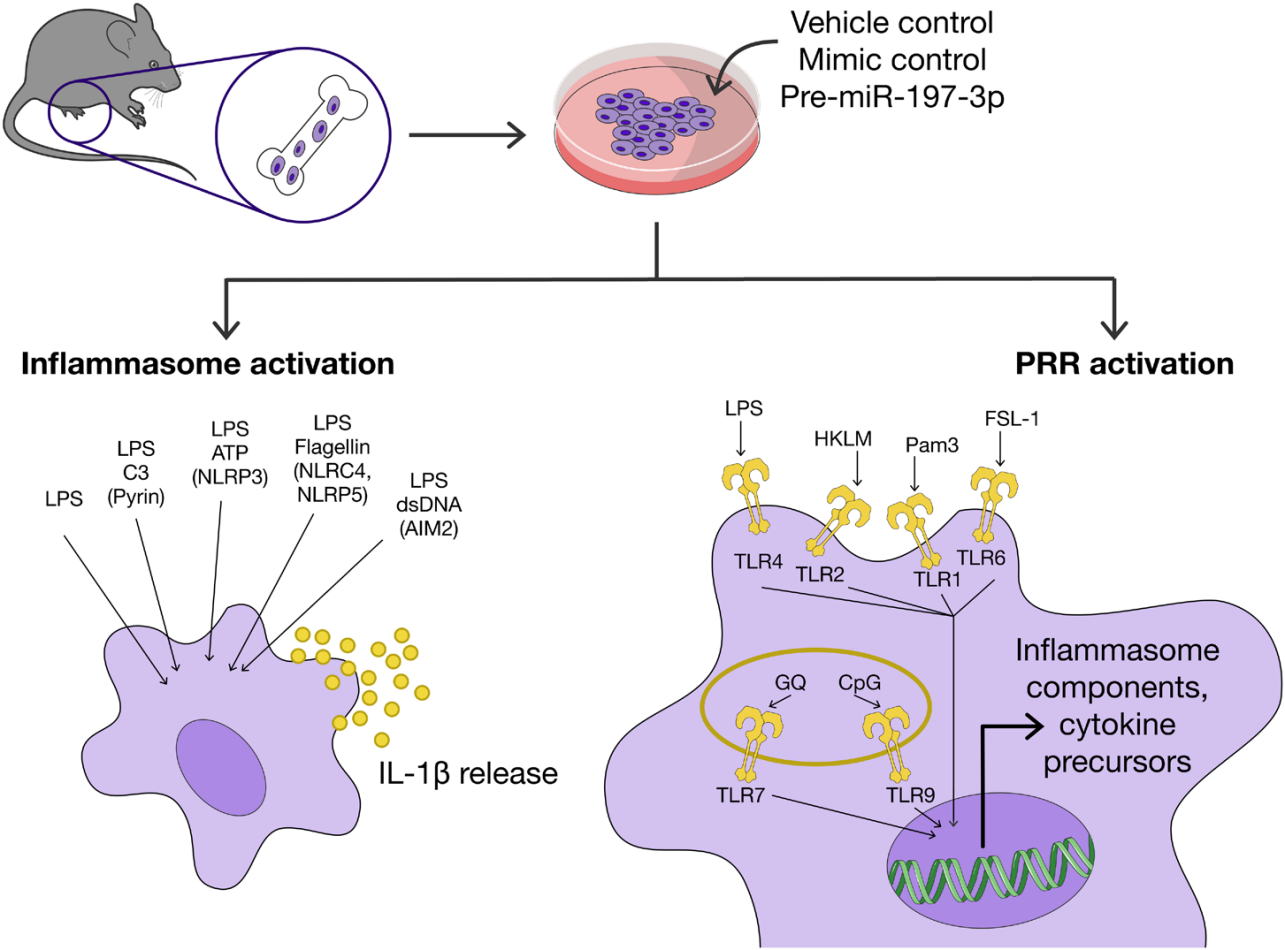
Experimental workflow of the study. Bone marrow–derived macrophages (BMDMs) from FMF KI mice were transfected with pre-miR-197-3p, mimic negative control, or vehicle control (Lipofectamine 2000). Cells were subsequently primed and stimulated using specific inflammasome activators. IL-1β levels in culture supernatants were quantified by ELISA. Expression levels of pro-IL-1β, ASC, NLRP3, and pyrin were assessed by immunoblot analysis. LPS, lipopolysaccharide; Pam3, Pam3Cys-Ser-(Lys)4 (TLR1/TLR2 agonist); CpGODN, Class A CpG ODN 2395 (TLR9 agonist); FSL-1, TLR2/TLR6 agonist; HKLM, heat-killed Listeria monocytogenes (TLR2 agonist); GQ, GQ single-stranded RNA (ssRNA) PolyU LyoVec (TLR7 agonist). Figure partly generated using Servier Medical Art under Creative Commons Attribution 3.0 Unported license.

**Figure 2.**
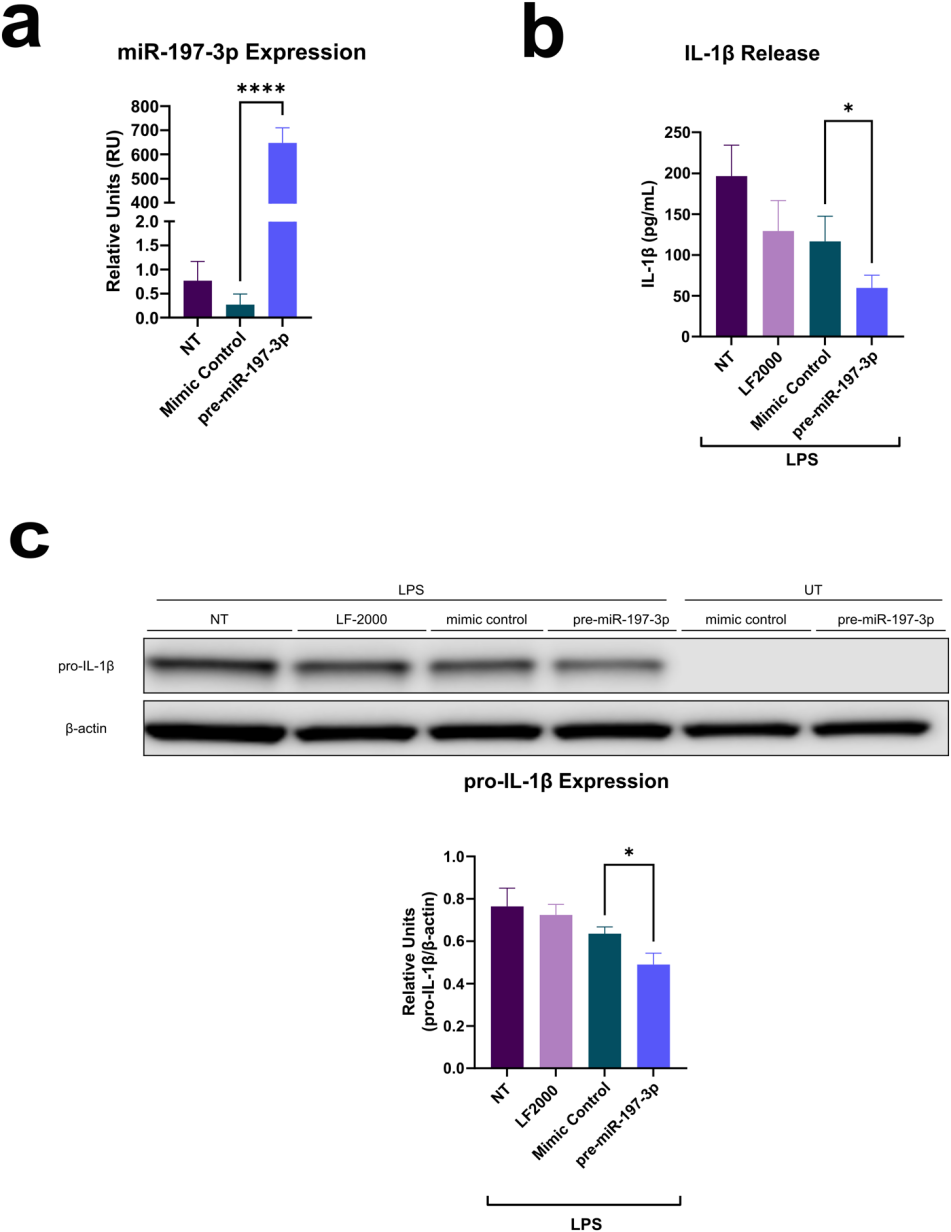
MiR-197-3p is associated with reduced IL-1β release in FMF KI mouse BMDMs. (a) RT-qPCR analysis of miR-197-3p expression in non-transfected cells or after transfection with pre-miR-197-3p or mimic negative control. (b) ELISA quantification of IL-1β secretion in culture supernatants following LPS stimulation. (c) Immunoblot analysis of pro-IL-1β expression in cell lysates with or without LPS treatment. Data are presented as mean ± SD from 3 biological replicates. Statistical analysis was performed using Student’s *t*-test or one-way ANOVA. *P ≤ .05; ****P ≤ .0001. NT, non-transfected; LF-2000, Lipofectamine-2000 vehicle control; LPS, lipopolysaccharide; UT, untreated.

The BMDMs isolated from FMF KI mice were used to assess the effects of miR-197-3p in the inflammatory process (Figure 1). Successful overexpression of pre-miR-197-3p in mouse BMDMs was confirmed by RT-qPCR (**: *P* ≤ .0001) (Figure 2A). Cells transfected with pre-miR-197-3p and stimulated with LPS showed a significant reduction in IL-1β release into the culture supernatant, as measured by ELISA (*: *P* ≤ .05) (Figure 2B). Pro-IL-1β expression induced by LPS was also significantly decreased in cells overexpressing pre-miR-197-3p (*P ≤ .05) (Figure 2C).

Activation of multiple inflammasome families, triggered by distinct stimuli and signaling pathways, leads to cleavage and secretion of IL-1β.

To identify the inflammasome pathway most closely associated with miR-197-3p-related changes in IL-1β release, secretion analyses were repeated using specific activators of the AIM2, NLRC4, NLRP3, and pyrin inflammasomes. Cells were treated with LPS together with the corresponding activators (dsDNA, flagellin, ATP, and C3 toxin).

**Figure 3.**
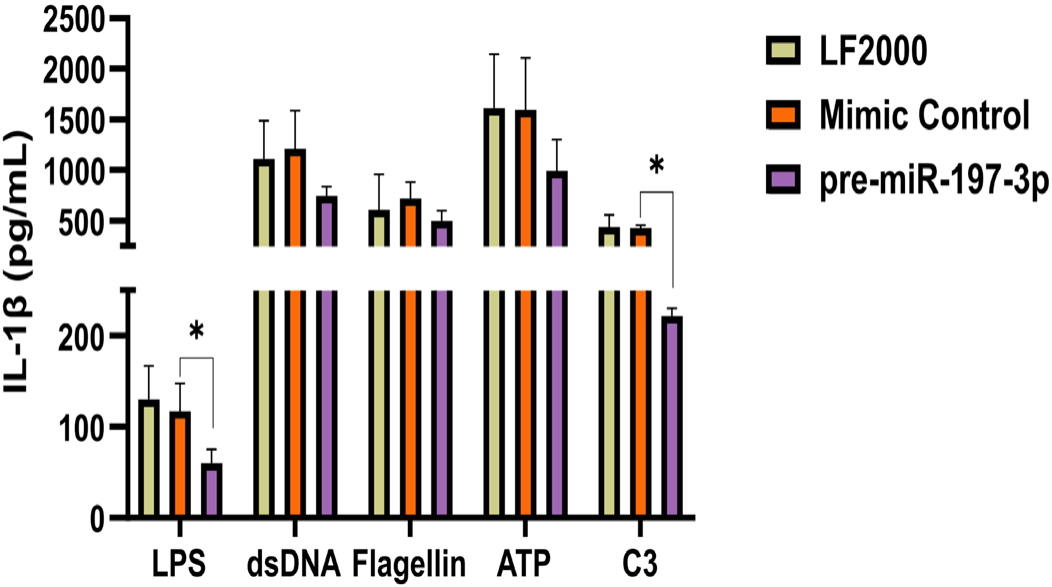
MiR-197-3p is associated with altered IL-1β release following inflammasome stimulation. IL-1β secretion was quantified by ELISA in culture supernatants of cells transfected with pre-miR-197-3p, mimic control, or vehicle control following inflammasome activation. Cells were primed with LPS for 3 hours prior to stimulation. AIM2 activation, dsDNA (1.25 μg/mL) with Lipofectamine 2000; NLRC4 activation: flagellin (0.5 μg/mL) with DOTAP; NLRP3 activation: ATP (5 mM, 30 minutes); Pyrin activation: C3 toxin (0.5 μg/mL, 6 hours). Data are presented as mean ± SD from 3 biological replicates. Statistical analysis was performed using Student’s *t*-test or one-way ANOVA. *P ≤ .05. LF-2000, Lipofectamine-2000 vehicle control.

The IL-1β secretion was significantly reduced in pre-miR-197-3p transfected cells stimulated with LPS alone (*P ≤ .05) and LPS + C3 toxin (*P ≤ .05), compared with mimic control cells (Figure 3). Activation of AIM2, NLRP3, and NLRC4 inflammasomes appeared to be largely preserved, as indicated by comparable IL-1β secretion and immunoblot profiles relative to mimic control cells.

**Figure 4.**
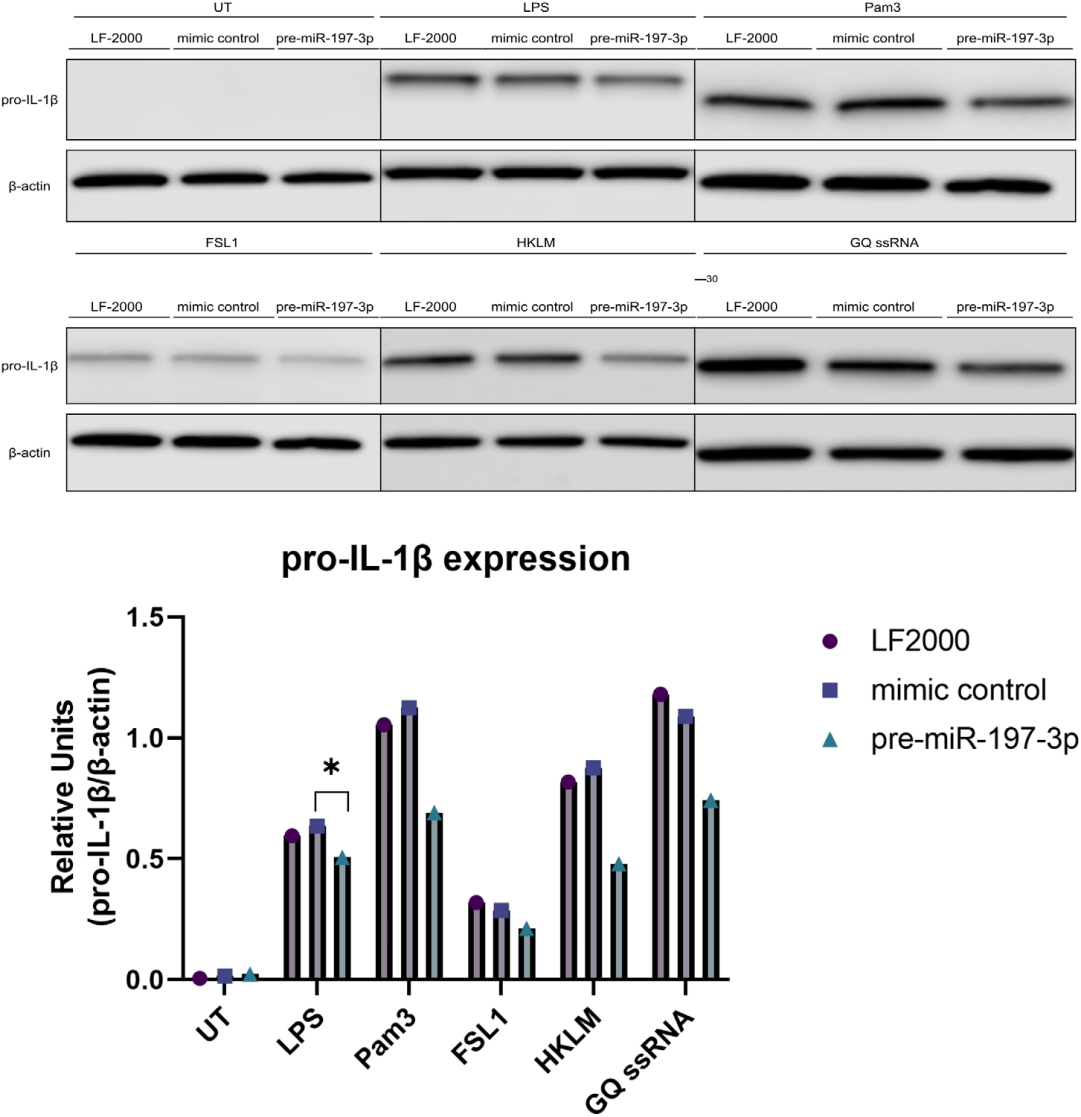
MiR-197-3p is associated with reduced pro-IL-1β expression following TLR agonist stimulation. Immunoblot analysis of pro-IL-1β expression in cell lysates transfected with pre-miR-197-3p, mimic control, or vehicle control after exposure to TLR agonists as indicated. Pam3: TLR1/TLR2 agonist; FSL-1, TLR2/TLR6 agonist; HKLM, TLR2 agonist; GQ: TLR7 agonist. Priming was performed for 3 hours in RPMI 1640 medium supplemented with 10% FBS and antibiotics. Data are presented as mean ± SD from 3 biological replicates. Statistical analysis was performed using Student’s *t*-test or one-way ANOVA. *P ≤ .05.

Inflammasome activation requires prior transcriptional priming, during which PAMP recognition by PRRs induces the expression of inflammasome components and cytokine precursors (Figure 1). In order to assess the effect of miR-197-3p on these upstream pathways, BMDMs were treated with various small-molecule activators of TLR family members and pro-IL-1β expression was evaluated with immunoblot (Figure 4). Pro-IL-1β expression was reduced in cells treated with Pam3 and HKLM in the presence of miR-197-3p overexpression.

**Figure 5.**
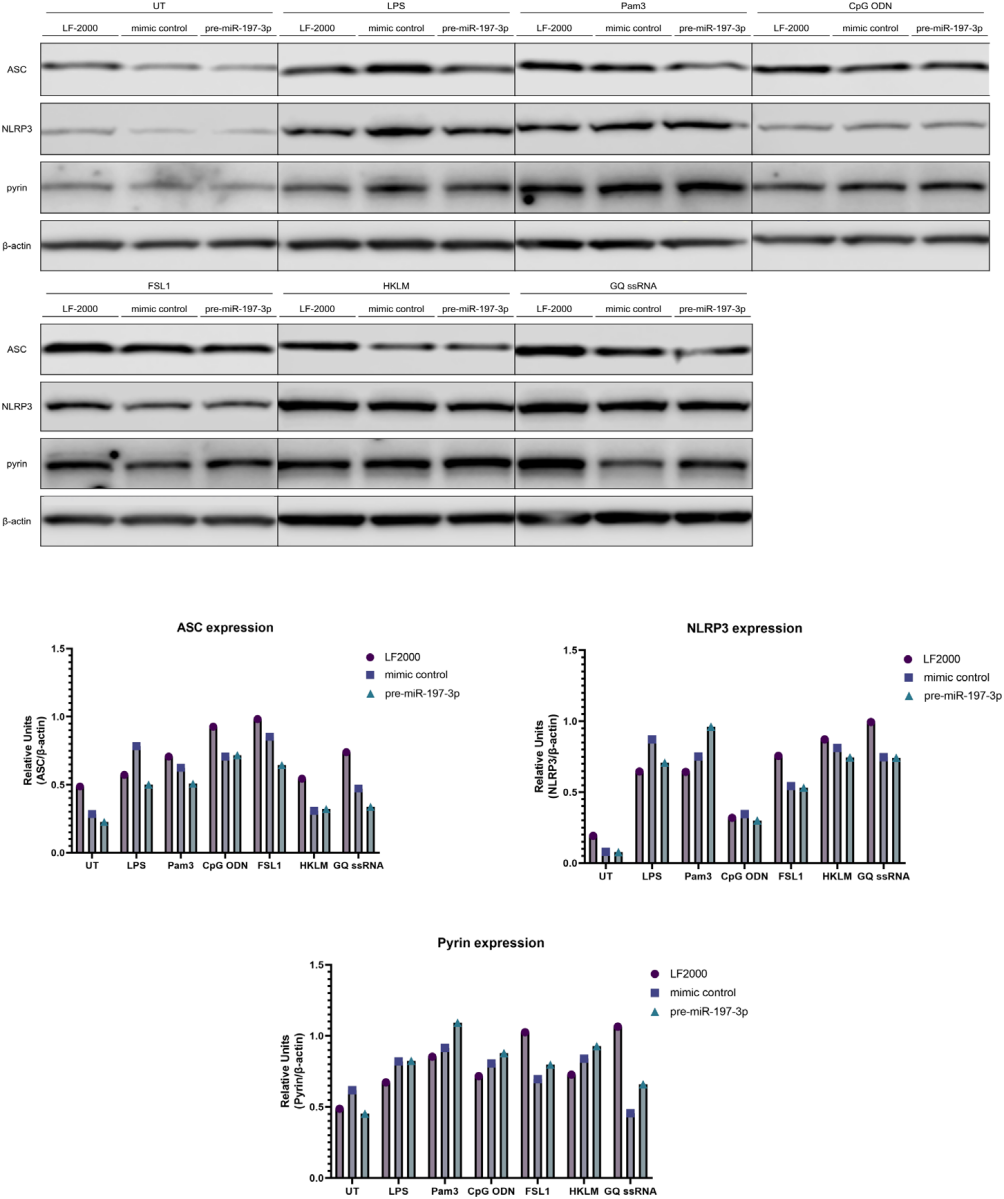
MiR-197-3p is associated with changes in inflammasome-related protein expression following TLR stimulation. Immunoblot analysis of pro-IL-1β expression in cells transfected with pre-miR-197-3p, mimic control, or vehicle control following stimulation with the indicated TLR agonists. UT, untreated; LF-2000, Lipofectamine-2000 vehicle control; LPS, lipopolysaccharide; Pam3, TLR1/TLR2 agonist; CpGODN, TLR9 agonist; FSL-1, TLR2/TLR6 agonist; HKLM, TLR2 agonist; GQ, TLR7 agonist. Priming was performed for 3 hours in RPMI 1640 medium supplemented with 10% FBS and antibiotics. Data are presented as mean ± SD from 3 biological replicates. Statistical analysis was performed using Student’s *t*-test or one-way ANOVA.

Expression of the inflammasome components ASC, NLRP3, and pyrin was further evaluated to assess the potential effects on priming (Figure 5). MiR-197-3p overexpression was associated with reduced ASC expression in cells treated with LPS, Pam3, GQ ssRNA, and FSL-1, but not with CpGODN or HKLM. NLRP3 expression was also reduced in response to miR-197-3p following LPS stimulation. Pyrin expression levels were not detectably altered under these conditions.

## Discussion

Mutations in the *MEFV* gene are the primary cause of FMF, an autosomal recessive inflammasomopathy. Recent studies have shown that pyrin-associated diseases linked to *MEFV* mutations may display inheritance patterns distinct from classical FMF.^15^^,^^16^ Nevertheless, genotype–phenotype correlations remain challenging, as individuals carrying identical mutations often exhibit heterogeneous clinical manifestations. These observations highlight the potential importance of epigenetic regulators, including miRNAs, in FMF pathophysiology.

In the previous studies, miR-197-3p was shown to participate in inflammatory regulation through targeting of IL1R1, suggesting a regulatory role in IL-1β–dependent inflammatory processes.^10^

In the present study, miR-197-3p overexpression was associated with decreased IL-1β release, predominantly following pyrin inflammasome activation. These findings suggest that miR-197-3p may preferentially influence pyrin inflammasome–related inflammatory responses rather than broadly suppressing inflammasome activity. Importantly, the present data do not directly demonstrate how miR-197-3p could affect pyrin inflammasome priming and activation but rather indicate a functional association at the level of IL-1β secretion.

Furthermore, miR-197-3p overexpression was associated with reduced pro-IL-1β expression in the presence of TLR2 agonists. However, this study does not directly establish how miR-197-3p modulates inflammasome priming or activation in FMF-KI macrophages, and the observed effects should therefore be interpreted as descriptive rather than mechanistically conclusive.

Several miRNAs have been linked with NLRP3 and noncanonical caspase-8 inflammasome activation and to target upstream signaling cascades including IL-1R, TLRs, IFNAR, and TNFR pathways.^17^^,^^18^ These miRNAs have also been shown to be dysregulated in other immune disorders.^19^ The present study represents one of the first investigations suggesting a possible association between miR-197-3p and pyrin inflammasome–related responses in FMF-KI macrophages.

Given that miR-197-3p directly targets IL1R1,^10^ it is not surprising that the overexpression of miR-197-3p is associated with decreased IL-1β secretion. But there are many other inflammasomes that play crucial roles in immune system regulation. For FMF disease, pyrin inflammasome is the major disease-causing inflammasome. In addition, it was demonstrated that in FMF KI mice models, mutant pyrin induces IL-1β activation through pyrin inflammasome.^14^ Therefore, the link between miR-197-3p and inhibition of pyrin inflammasome may be important for understanding the disease pathogenesis.

The results demonstrated that IL-1β secretion was decreased significantly in miR-197-3p transfected cells compared to mimic control transfected cells. It was demonstrated that the level of inflammasome components did not change with miR-197-3p overexpression with priming agents. These findings suggest that the threshold for inflammasome activation is not related to the amount of proteins produced, but rather to the function of the inflammasome itself. In this context, secreted IL-1β levels may represent the most relevant readout of inflammasome-related functional outcomes.

Based on the observed association with pyrin inflammasome activation, potential upstream priming pathways were next explored. Pro-IL-1β expression was affected by miR-197-3p in the presence of LPS, Pam3, and HKLM. Because Pam3 and HKLM both signal through TLR2, this receptor may represent a potential upstream regulatory node influenced by miR-197-3p during inflammasome priming.

Despite the strengths of this study, several limitations should be acknowledged. First, the experiments were conducted exclusively in BMDMs isolated from an FMF knock-in mouse model, which may not fully recapitulate the complexity of inflammatory responses observed in human FMF patients.

Second, although miR-197-3p was shown to modulate IL-1β secretion and pro–IL-1β expression, the present study does not directly elucidate the precise molecular mechanisms by which miR-197-3p influences inflammasome priming and activation beyond its established targeting of IL1R1. In addition, potential direct or indirect interactions between miR-197-3p and upstream TLR signaling components were not functionally validated.

Finally, the lack of in vivo therapeutic intervention studies limits the ability to draw definitive conclusions regarding the translational potential of miR-197-3p–based approaches. Future studies incorporating in vivo models and mechanistic target validation will be necessary to address these limitations.

In conclusion, this study demonstrates that miR-197-3p overexpression is associated with reduced IL-1β release following pyrin inflammasome activation in FMF knock-in mouse macrophages. The selective nature of this association effect of miR-197-3p on the pyrin inflammasome, rather than on other inflammasome pathways, suggests a targeted regulatory role that is particularly relevant to FMF pathogenesis.

Furthermore, the reduction of pro–IL-1β expression in response to TLR2 agonists indicates that miR-197-3p may modulate inflammasome priming through upstream innate immune signaling pathways.

Although *MEFV* mutations are the primary genetic drivers of FMF, the variability in clinical phenotype among individuals with identical genotypes highlights the importance of epigenetic regulators such as miRNAs. In this context, miR-197-3p emerges as a potential modulator of pyrin inflammasome–dependent inflammation rather than a general suppressor of inflammasome activity.

While the present findings do not support immediate therapeutic application, they provide a conceptual framework suggesting that miR-197-3p–based modulation could represent a complementary strategy for controlling IL-1β–driven inflammation in FMF and potentially other pyrin-associated autoinflammatory disorders. Further mechanistic and in vivo investigations will be required to assess the translational relevance of these observations.

## Data Availability

The data that support the findings of this study are available on request from the corresponding author.
